# Screening for *EGFR* and *KRAS* Mutations in Endobronchial Ultrasound Derived Transbronchial Needle Aspirates in Non-Small Cell Lung Cancer Using COLD-PCR

**DOI:** 10.1371/journal.pone.0025191

**Published:** 2011-09-19

**Authors:** George Santis, Roger Angell, Guillermina Nickless, Alison Quinn, Amanda Herbert, Paul Cane, James Spicer, Ronan Breen, Emma McLean, Khalid Tobal

**Affiliations:** 1 Division of Asthma, Allergy & Lung Biology, King's College London, London, United Kingdom; 2 Molecular Oncology Diagnostics Unit, Guy's & St Thomas' National Health Service Foundation Trust, London, United Kingdom; 3 Department of Respiratory Medicine, Guy's & St Thomas' National Health Service Foundation Trust, London, United Kingdom; 4 Department of Pathology, Guy's & St Thomas' National Health Service Foundation Trust, London, United Kingdom; 5 Division of Cancer Studies, King's College London, London, United Kingdom; Comprehensive Pneumology Center, Germany

## Abstract

*EGFR* mutations correlate with improved clinical outcome whereas *KRAS* mutations are associated with lack of response to tyrosine kinase inhibitors in patients with non-small cell lung cancer (NSCLC). Endobronchial ultrasound (EBUS)-transbronchial needle aspiration (TBNA) is being increasingly used in the management of NSCLC. Co-amplification at lower denaturation temperature (COLD)–polymerase chain reaction (PCR) (COLD-PCR) is a sensitive assay for the detection of genetic mutations in solid tumours. This study assessed the feasibility of using COLD-PCR to screen for *EGFR* and *KRAS* mutations in cytology samples obtained by EBUS-TBNA in routine clinical practice. Samples obtained from NSCLC patients undergoing EBUS-TBNA were evaluated according to our standard clinical protocols. DNA extracted from these samples was subjected to COLD-PCR to amplify exons 18–21 of *EGFR* and exons two and three of *KRAS* followed by direct sequencing. Mutation analysis was performed in 131 of 132 (99.3%) NSCLC patients (70F/62M) with confirmed lymph node metastases (94/132 (71.2%) adenocarcinoma; 17/132 (12.8%) squamous cell; 2/132 (0.15%) large cell neuroendocrine; 1/132 (0.07%) large cell carcinoma; 18/132 (13.6%) NSCL-not otherwise specified (NOS)). Molecular analysis of all *EGFR* and *KRAS* target sequences was achieved in 126 of 132 (95.5%) and 130 of 132 (98.4%) of cases respectively. *EGFR* mutations were identified in 13 (10.5%) of fully evaluated cases (11 in adenocarcinoma and two in NSCLC-NOS) including two novel mutations. *KRAS* mutations were identified in 23 (17.5%) of fully analysed patient samples (18 adenocarcinoma and five NSCLC-NOS). We conclude that EBUS-TBNA of lymph nodes infiltrated by NSCLC can provide sufficient tumour material for *EGFR* and *KRAS* mutation analysis in most patients, and that COLD-PCR and sequencing is a robust screening assay for *EGFR* and *KRAS* mutation analysis in this clinical context.

## Introduction

Epidermal growth factor receptor (EGFR) is a member of the ErbB receptor family, a key regulator of epithelial cell proliferation [1]. EGFR consists of an extracellular domain, a transmembrane region and a cytoplasmic catalytic region that includes the tyrosine kinase domain [1]. Excessive EGFR signaling upsets the balance between cell growth and apoptosis contributing to tumourigenesis in a wide variety of solid tumours including non-small cell lung cancer (NSCLC) [2]. This can arise from overexpression of EGFR, its signaling partners, or two of its ligands, EGF and TGF-α [3], [4]. Constitutive activation of EGFR tyrosine kinase activity can be brought about by somatic mutations in the tyrosine kinase domain of EGFR [5], [6], [7]. Retrospective and prospective studies in Asian and European patients with NSCLC have shown that the presence of *EGFR* mutations in exons 18–21 correlates with superior clinical outcome to EGFR tyrosine kinase inhibitors gefitanib and erlotinib [8], [9], [10]. Most NSCLC-specific *EGFR* mutations are either a single amino acid substitution at codon 858 (Leucine to Argine; L858R), or deletion mutations in exon 19 that affect the conserved LREA motif [11]. These mutations are found in a minority of Caucasian patients with NSCLC but as many as 60% of East Asians with adenocarcinoma [8], [12], [13], [14]. A separate group of *EGFR* mutations is associated with primary as well as acquired resistance to erlotinib and gefitinib, and these cluster in exon 20 of the *EGFR* gene [15], [16].

Some NSCLC also harbour mutations in Kirsten rat sarcoma viral oncogene homolog *(KRAS)* encoding a GTPase downstream of EGFR [17], [18], [19]. These mutations cluster in exon two of *KRAS*, occur in 15–30% of unselected NSCLC [20], and appear to be mutually exclusive to *EGFR* mutations in NSCLC [21]. It has been suggested that mutations in *KRAS* are associated with *de novo* resistance to gefitinib and erlotinib [21]. Unlike *EGFR* mutations, which are a positive prognostic factor, *KRAS* mutations in resected NSCLC were associated with shorter overall survival than those with *EGFR* mutations [17], [18], [19]. Taken together current evidence suggests that *EGFR* and *KRAS* mutations define distinct subgroups of NSCLC patients, with different responses to EGFR- targeted therapies.

Most patients with NSCLC present at an advanced stage and pathological diagnosis is often made from small-sized bronchoscopic, transthoracic core biopsies or cytological samples. Most genetic mutation analyses rely on the polymerase chain reaction (PCR) for amplification of target sequences. Unlike standard PCR, co-amplification at lower denaturation temperature-PCR (COLD-PCR) preferentially amplifies mutant sequences and therefore increases the sensitivity of detecting genetic mutations [22]. This is particularly important in analysing the presence of genetic mutations in solid cancer tissues, where tumour cells may be admixed with stromal and other non-malignant tissue. Since it was first described, COLD-PCR has been shown to be superior to conventional PCR in a number of applications designed to detect mutations in mixed samples [22], [23], [24], [25].

Endobronchial ultrasound (EBUS)-transbronchial needle aspiration (TBNA) is a recently developed technique that allows ultrasound-guided aspiration of mediastinal and hilar lymph nodes and masses. Increasing data supports its use in lung cancer diagnosis and staging as an alternative to mediastinoscopy [26], [27], [28], [29], [30], however there are concerns that these small cytological samples may provide insufficient tumour material for molecular diagnosis, an area of increasing importance in NSCLC management. This is reflected in a recently published consensus statement on *EGFR* mutation testing which recommends that tissue biopsy samples should be used in preference to cytological samples whenever possible, until further research establishes the reliability of mutational data obtained from cytological samples [31]. Here we address this question by screening for *EGFR* and *KRAS* mutations in 193 EBUS-TBNA derived cytology samples from metastatic lymph nodes in 132 patients with NSCLC in routine clinical practice using a single assay based on the principles of COLD-PCR and direct sequencing.

## Results

### *EBUS-TBNA*

132 patients diagnosed with NSCLC using EBUS-TBNA between May 2009 and February 2011 (125 Caucasian, four Asian and three British Black) were included in this study. All patients (n = 65) with NSCLC irrespective of histological sub-type between May 2009 and February 2010 were included in this study. The remaining 67 patients (March 2010–February 2011) represent consecutive patients with NSCLC, non-squamous sub-type. Patient clinical characteristics and disease stage are shown in Table 1. None of the patients had received treatment prior to the procedure. Aspirates were obtained from 193 lymph nodes from stations two to 11 (short axis diameter was 1.2+/−0.5 cm) in 132 patients (Table 1). The median number of passes per lymph node station was 4.6 (range: 1–10); this is similar to the median number of passes per lymph node station (3.8) obtained in 972 patients investigated at our centre by EBUS-TBNA between February 2008 and February 2011 (unpublished observations).

**Table 1:** Patient Characteristics.

**Age**	
*Mean*	65.5 years
*Range*	*45–81 years*
**Male**	*53 (%)*
**Female**	*47 (%)*
**Tumour type**	
*Adenocarcinoma*	*94/132 (71.2%)*
*Squamous cell*	*17/132 (12.8%)*
*NSCLC-NOS* *	*18/132 (13.6%)*
*Large cell neuroendocrine*	*2/132 (0.15%)*
*Large cell Carcinoma*	*1/132 (0.07%)*
**Lymph Node Stations sampled by EBUS-TBNA** +	
*2R*	*12/193 (6%)*
*2L*	*4/193 (2%)*
*4R*	*55/193 (29%)*
*4L*	*23/193 (12%)*
*7*	*48/193 (25%)*
*10R*	*20/193 (10%)*
*10L*	*10/193 (5%)*
*11R*	*6/193 (3%)*
*11L*	*15/193 (8%)*

*Refers to not otherwise specified.

+ Refers to delineation of lymph node stations by endobronchial ultrasound (EBUS) was based on the new International Association of Study of Lung Cancer (IASLC) lymph node map.

### Morphological diagnosis and immunoprofile

Immunohistochemistry was successfully performed in 131 of 132 patients. Insufficient material was available from one patient. Histological type was determined by a combination of morphology (cytological slides and cellblock sections) and immunohistochemical profile. In the case of adenocarcinoma, diagnosis was supported by expression of TTF-1, CK7 or BerEP4 and negativity for CK5 and p63. Cytomorphology and expression of CK5 and p63 favoured squamous cell carcinoma diagnosis, while expression of neuroendocine markers (CD56, chromogranin and synaptophysin) and appropriate morphology established the diagnosis of large cell neuroendocrine carcinoma. Undifferentiated NSCLC by morphology that also lacked expression of differentiation markers CK5, CK7, CD56, TTF-1, p63, BerEP4 resulted in the diagnosis of NSCLC not otherwise specified (NSCLC-NOS). Based on these criteria, 94 of the 132 patients (71.2%) were diagnosed with adenocarcinoma, 17 (12.8%) with squamous cell carcinoma, two with large neuroendocrine cell carcinoma (0.15%), one with large cell carcinoma (0.07%), and 18 (13.6%) with NSCLC-NOS (Table 1).

### Mutation Analysis

The COLD-PCR and sequencing protocol was optimised to amplify and sequence exons 18 to 21 of *EGFR* and codons 12, 13 and 61 of *KRAS* in order to detect *EGFR* and *KRAS* mutation with sensitivity of 5–10% (mutation frequencies of 10% were detected in all COLD-PCR runs, whereas mutation frequencies of 5% were detected in two of every three runs) compared to mutation sensitivity of 30% for our standard-PCR protocol.

One of the 132 samples failed to amplify target DNA and therefore *EGFR* and *KRAS* mutation analysis was performed in 131 of 132 samples (99.3%). The patient sample that failed DNA amplification contained only 100 cells/section [32]. The COLD-PCR protocol successfully amplified exons 18–21 in 126 of 131 patients (95.5%) in whom DNA was available. Amplification of exon 21 failed in three patient samples (two adenocarcinomas and one squamous cell carcinoma); one of these samples also failed amplification of exon 20. Two additional patient samples failed amplification of exon 18. Sequencing was successful for all amplified sequences; therefore complete molecular analysis of all four *EGFR* target exons was available in 126 of the 132 patients (95.4%) and partial molecular analysis in 131 of 132 patients included in this study (99%).

Using COLD-PCR we were able to detect *EGFR* mutations in 13 of 126 patients (10.3%) in whom full molecular analysis was available (Table 2). One patient sample contained two exon 21 mutations (Table 3). Repeating the COLD-PCR and sequencing protocol from a second cellblock independently confirmed all mutations. Mutations were almost exclusively found in adenocarcinoma sub-type (11 of 13; 85%; p<0.001). One large in-frame deletion in exon 19 and the L858R mutation were detected in two patients with NSCLC-NOS. No *EGFR* mutations were detected in 17 squamous cell carcinomas between May 2009 and February 2010; mutation analysis was subsequently performed only in patients diagnosed with NSCLC non-squamous histology. *EGFR* mutations were identified in 11 of 89 (12.3%) adenocarcinomas and 13 of 110 (12%) non-squamous histology. The L858R mutation accounted for four of 13 (31%). We identified only one in-frame deletion in exon 19 (Δ2481–2495) (Table 3). We also identified two novel *EGFR* mutations; both were single amino acid substitutions, one in exon 19 (V760M) and another in exon 20 (H805L). There was one complex mutation (L833V + L858R). The possibility exists that novel mutations detected in this study are artefacts; this has been linked to PCR of formalin-embedded tissue [33], [34]. Moreover, COLD-PCR as well as a standard-PCR protocol is susceptible to polymerase-induced errors. In our study AmpliTaq Gold was used to amplify target sequences, in contrast to high fidelity Taq polymerase used by Li *et al.* [22], [23], [24] Using high fidelity Taq polymerase could avoid the possibility of PCR enrichment of PCR errors. However, it is unlikely that the novel mutations detected in our study are due to COLD-PCR errors, as all mutations were confirmed in separate reactions and no mutations were detected in wild type DNA that was used as negative control in all reactions. This would also suggest that AmpliTaq Gold did not enrich amplicons with artificial mutations.

**Table 2:** Frequency of *EGFR* and *KRAS* mutations in metastatic lymph nodes in NSCLC*.

Tumour type	*EGFR* mutations (%)	*KRAS* mutations (%)
Adenocarcinoma	11/89 (12.3%)	18/93 (19%)
NSCLC-NOS+	2/18 (11.1%)	5/18 (27.7%)
Large cell neuroendocrine	0/2	0/2
Large cell carcinoma	0/1	0/1
Squamous cell	0/16	0/16
Non-squamous	13/110 (12%)	23/114 (20%)

*Refers to data for fully analysed patient samples.

+ Refers to not otherwise specified.

**Table 3:** *EGFR* and *KRAS* mutations detected by COLD-PCR.

	*EGFR*		Number of cases
Adenocarcinoma	G719A+	Exon 18	2
Adenocarcinoma	L747P+	Exon 19	1
NSCLC-NOS∧	2481-2495del15	Exon 19	1
Adenocarcinoma	P733S+	Exon 19	1
Adenocarcinoma	V760M*	Exon 19	1
Adenocarcinoma	H805L*	Exon 20	1
Adenocarcinoma	2319 insertion CAG2320+	Exon 20	1
Adenocarcinoma	L858R+	Exon 21	2
Adenocarcinoma	L833V++L858R+	Exon 21	1
Adenocarcinoma	L861E+	Exon 21	1
NSCLC-NOS∧	L858R+	Exon 21	1

	*KRAS*		
Adenocarcinoma	G12C	Exon 2	13
Adenocarcinoma	G12V	Exon 2	4
Adenocarcinoma	G61H	Exon 3	1
NSCLC-NOS∧	G12C	Exon 2	3
NSCLC-NOS∧	G12V	Exon 2	1
NSCLC-NOS∧	G61H	Exon 3	1

+ Refers to known *EGFR* mutations.

*Refers to novel *EGFR* mutations.

^∧^Refers to not-otherwise specified.

We also identified the less common *EGFR* mutations G719A, P733S, L747P and L861Q. Another uncommon mutation (L833V) was found together with L858R mutation. MassArray (Sequenom Inc) and Scorpion amplified refractory mutation system (SARMS) (DsX *EGFR* PCR mutation analysis kit; QIAGEN) technologies would not have detected mutations P733S, L747P, V760M, H805L and L833V.

*KRAS* mutations analysis was successful in 130 of 132 tumours (98.4%). One sample that gave uninformative sequence also failed *EGFR* mutation analysis due to paucity of tumour material. Single amino acid substitutions involving codons 12, 13 and 61 of *KRAS* were identified in 23 of 130 NSCLC (17.7%) overall (18 of 93 adenocarcinomas (19%) and five of 18 NSCLC-NOS (27.7%)) (Table 3). None were found in patients with squamous cell tumours or in patients harbouring *EGFR* mutations.

**Figure 1:**
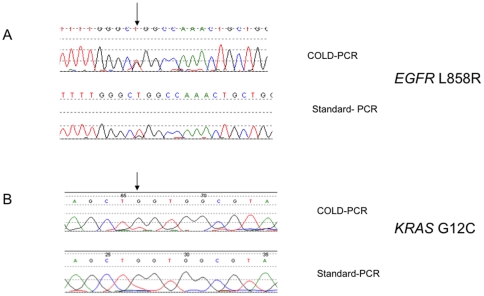
Examples of comparative analysis of COLD-PCR *vs* standard-PCR. Sequencing electrogramme of comparative analysis of COLD-PCR and standard-PCR amplification of *EGFR* exon 19 and *KRAS* exon 2. A: upper and lower panels are COLD and standard PCR amplification of exon 21 of *EGFR* from EBUS-derived aspirates from lymph nodes infiltrated by metastatic lung adenocarcinoma respectively. A shows substitution of thymidine (T) by cytosine (C) in exon 21 of *EGFR* to generate L858R mutation that is evident in the COLD-PCR amplification reaction (arrow) as well as the standard PCR reaction. The mutation peak is more clearly visible in the COLD-PCR (upper panel) compared to standard-PCR reaction (lower panel). B upper and lower panels are COLD and standard PCR amplification of *KRAS* exon 2. The upper panel shows substitution of guanine (C) by thymidine (T) to generate G12C mutation that was detected by COLD-PCR amplification (arrow). The mutation was not evident in the standard-PCR reaction (lower panel).

Previous studies and our own validation experiments have shown increased sensitivity of COLD-PCR compared to standard PCR protocols [22], [23], [24], [25], [35]. Here we also performed a limited comparison of the ability of COLD-PCR to detect *EGFR* and *KRAS* mutations in 25 EBUS-derived adenocarcinoma cytological aspirates with that of standard-PCR. These samples were also analysed in parallel by COLD-PCR and SARMS (DxS EGFR PCR kit, QIAGEN) according to manufacturer instructions. We found standard-PCR and subsequent sequencing detected all *EGFR* mutations that had been detected by COLD-PCR (L858R, Δ2481–2495 and H805L). The mutation peak was more clearly visible following COLD-PCR amplification as shown for the L858R mutation (Figure 1A). Standard-PCR failed to detect the *KRAS* G12C mutation (Figure 1B). This difference in mutation detection between COLD-PCR and standard PCR was not significant (p = 0.5). Using SARMS, we found no additional *EGFR* mutations among these EBUS-derived adenocarcinoma aspirates. SARMS also confirmed the L858R mutation that had been originally identified by COLD-PCR.

## Discussion

Here we report on the feasibility of using COLD-PCR to screen for *EGFR* and *KRAS* mutations in 132 patients that had been sampled by EBUS-TBNA according to our standard clinical protocols. This represents the largest cohort of such patients reported. We demonstrate complete evaluation of exons 18 to 21 in 95.5% of *EGFR* and exons two and three in 98.4% of *KRAS* amongst EBUS-TBNA aspirates. Our results compare favourably with three previous studies that screened EBUS-derived aspirates for *EGFR* mutations. Garcia-Olive *et al* and Nakajima *et al* successfully analysed exons 19 and 21 of *EGFR* in 72% (26/36) and 93% (43/46) of patient samples respectively [36], [37]. Schuurbier *et al* successfully analysed 77% of all samples by standard PCR and sequencing of exons 18–21 of *EGFR* [38]. We sampled broadly similar sized lymph nodes as those evaluated in the other three studies and performed a median of 4.5 passes per lymph node sampled; this number of passes is similar to the three-to-four passes per node advised to establish the diagnosis of malignancy [28], [30]. Results from this and previous studies therefore suggest that EBUS-TBNA can provide sufficient tumour material for *EGFR* and *KRAS* mutation analysis in routine clinical practice thus avoiding the need for more invasive surgical sampling in these patients.

There are currently a number of methods that have been developed to screen for *EGFR* mutations in NSCLC samples where mutant DNA represents only a fraction of total purified DNA [7]. Some assays such as SARMS and MassArray screen for specific mutations with sensitivities of 1% and 10% respectively. Other strategies that rely on techniques such as high resolution melting and denaturing high performance liquid chromatography detect most mutations without specifying the precise amino acid substitution [7]. In some studies, microdissection of tumour DNA from tissue samples was performed prior to DNA amplification [39]. There is currently no general agreement on which of these represents the best method for mutation analysis in NSCLC [31]. However, strategies based on DNA amplification and direct sequencing are the most comprehensive as they can screen not only for known but also novel mutations. It is recommended that at least 30% tumour cells need to be present with more than 10% mutant DNA for efficient mutation screening relying on standard PCR and sequencing protocols [7].

The COLD-PCR assay used in this study detected *EGFR* and *KRAS* mutations present in tumour DNA comprising as little as 5–10% of total sample DNA. It is likely that, by employing a single COLD-PCR critical denaturation temperature for some of the amplicons tested there is a substantial enrichment (as Fig. 1 shows) while for others there is little or no enrichment. The sensitivity so defined of our COLD-PCR assay could have therefore been further improved to detect mutation frequencies of less than 5% had we developed an amplicon-specific assay using optimal heteroduplex annealing and denaturing temperatures for each amplicon. Sensitivity could be further enhanced by utilising more sensitive amplicon-specific COLD-PCR assays such as that described by Galbiati *et al* [40], or the Improved and Complete Enrichment-COLD-PCR (*ice*-COLD-PCR) platform that can detect mutation frequencies as low as 0.1%, [41]. It is our view however that utilising amplicon-specific assays is unlikely to be feasible for routine diagnostic use in the detection of multiple mutations and may best be employed when tumour cell content is lower than the 5–10% sensitivity threshold of our current assay. Interestingly, we recently found this to be an uncommon clinical scenario [32]. For example, we found that the median tumour cell count in EBUS-derived lymph node aspirates was 2525 (range 65-39800) and the median percentage tumour was 70% (range 10–95%). This was comparable to the yield from bronchoscopic biopsies and superior to the yield from computer tomography-guided needle biopsies of peripheral primary lung tumours. In fact the percentage tumour content in all sample types studied was 5% or higher [32]. As EBUS-derived clinical samples can be composed of less than 30% tumour cells, standard-PCR may not be sufficiently sensitive to detect sufficiently sensitive to detect mutations in these samples. In support of this, a limited comparison of COLD-PCR *vs* standard-PCR showed that standard-PCR failed to detect one of four mutations identified by COLD-PCR, which also showed higher mutation peaks (Figure 1A & 1B). Previous studies have also shown increased sensitivity of COLD-PCR compared to standard PCR protocols [22], [23], [24], [25], [35]. As COLD-PCR has no additional cost, we favour its use to standard-PCR to screen for *EGFR* and *KRAS* mutations in these clinical samples.

We found that the frequency of *EGFR* mutations in lung adenocarcinomas and non-squamous NSCLC was broadly in keeping with results from two previous large studies in European patients with lung adenocarcinomas that found *EGFR* mutations in 10% and 16.6% of patients, with exon 19 deletions representing 46% and 62% of all *EGFR* mutations [12], [14]. Two of the mutations (V760M and H805L) detected in our patient cohort were novel and two others (P733S, L747P) would not have been detected by SARMS (DX Quiagen) or by MassArray (Sequenom Inc) assays. Distinguishing novel *EGFR* mutations that are clinically relevant from those that are functionally silent or artefacts is clearly important, particularly as diverse responses to EGFR tyrosine kinase inhibitor (TKI) therapy of patients with NSCLC harbouring uncommon *EGFR* mutations were recently reported [42]. The less common G719A and L861Q mutations that were found in our patient cohort were shown to be sensitive to EFFR TKI therapy and are therefore clinically significant [42]. The L747P mutation has been linked to poor responsiveness to EGFR TKI inhibitor therapy, whilst another mutation at the same codon (L747S) has been linked to acquired resistance to TKI therapy. Exon 20 insertions, such as the 2319insertionGAC2320 found in our cohort, are also linked to poor response to EGFR TKI therapy [43]. We also identified one doublet mutation (L833V combined with L858R). Doublet mutations accounted for 6% of *EGFR* mutations, with approximately half of these occurring at five codons [44]. It is interesting however that L833V in combination with the H835L exon 21 mutation has been linked to favourable response to gefitinib [45]. We have no information regarding the responsiveness of our H833V+L858R mutation to TKI therapy.

We found only one deletion in exon 19 that accounted for 7% of all *EGFR* mutations. This is significantly lower (p<0.01) than the 36% frequencies of *EGFR* exon 19 deletions in NSCLC primary tumour specimens analysed by COLD-PCR and direct sequencing of exons 18–21 in our institution (unpublished). This observation raises the possibility that exon 19 deletions may be underrepresented in metastatic lymph nodes compared to primary tumours. Park *et al* reported discordance between primary tumour and lymph node metastases in NSCLC particularly for mutations in exon 19 [46]. Moreover, Nakajima *et al* reported only one exon 19 deletion among 11 (9.9%) *EGFR* mutations in 43 EBUS-TBNA metastatic lung adenocarcinomas in East-Asian patients [37], whereas in primary lung adenocarcinomas exon 19 deletions account for as much as 53% of mutations in East Asian patients [47]. Loss of *EGFR* mutations in metastatic lung adenocarcinomas compared to primary tumours has also been reported [48]. Larger prospective studies matching analysis of primary tumour and lymph node metastases are required to evaluate whether *EGFR* exon 19 deletions, or other mutations, are underrepresented in metastatic lymph nodes either at the time of diagnosis or in response to treatment.

In this study we also assessed EBUS-derived needle aspirates for *KRAS* mutations using COLD-PCR and found these in 19% of lung adenocarcinomas and 27.7% of NSCLC-NOS. COLD-PCR was previously shown to enhance *KRAS* mutation detection sensitivity compared to ordinary PCR, in a variety of clinical samples [25]. The frequency of *KRAS* mutations in the EBUS-TBNA samples analysed in this study is in keeping with previous studies that reported *KRAS* mutation frequency of up to 22%, predominantly in adenocarcinomas [20] Importantly, *KRAS* mutations are associated with lack of response to EGFR inhibitor therapy in NSCLC [21]. Taken together, our results demonstrate that by combining *EGFR* and *KRAS* mutation analysis in NSCLC patients with non-squamous cell histology, decisions on appropriateness of EGFR TKI therapy can be made in 27% of our patient cohort.

We conclude that EBUS-TBNA of mediastinal lymph nodes infiltrated by NSCLC can provide sufficient tumour material for *EGFR* and *KRAS* mutation analysis in the great majority of patients without the need to resort to more invasive surgical mediastinoscopy or mediastinotomy. We also conclude that COLD-PCR and sequencing protocols should be considered as a potential screening assay for multiple *EGFR* and *KRAS* mutation analysis in this clinical context. The ability to detect novel *EGFR* mutations, as we demonstrated in this study, may also prove useful in screening for acquired *EGFR* resistance mutations, an issue of emerging clinical importance in NSCLC. Serial sampling and assessment of tumour tissue obtained contemporaneously are increasingly recognised as important in the clinical use of EGFR-targeted therapies. EBUS-TBNA is a safe and minimally invasive technique that is likely to be eminently applicable in this context.

## Materials and Methods

### Ethics Statement

This was an observational study performed according to our standard clinical protocols. All patients gave their written consent to undergo EBUS-TBNA and for the sampled material to be analysed according to approved clinical protocols. EBUS-TBNA was approved as a new investigational procedure by Guy's & St Thomas' Hospital Clinical Governance Committee and is part of the standard of care of patients with NSCLC. COLD-PCR is the approved method for *EGFR* and *KRAS* mutation analysis at our institution. *EGFR* and *KRAS* mutation analysis is part of the standard of care of patients with NSCLC at our institution.

### EBUS-TBNA

EBUS-TBNA was performed by two consultants in Respiratory Medicine using a bronchoscope with integrated linear ultrasound probe (Olympus 260F) and the C200 ultrasound processor in 128 patients and the alpha five-ultrasound processor in four. The procedure was performed using conscious sedation. 22G needle was used to aspirate each node. One air-dried and one alcohol-fixed conventional smear was prepared from each pass by a biomedical scientist and needle washings were rinsed in balanced salt solution: Aqsia™ (Bausch & Lomb, Kingston-Upon-Thames, UK). Air-dried smears were stained with Hemacolor™ (Merck Chemicals Ltd, Nottingham, UK) for immediate assessment and alcohol-fixed slides retained for later Papanicoloau staining. On-site evaluation (ROSE) by a consultant cytopathologist provided real-time assessment of the aspirates and triage of cell suspensions for cellblocks, flow cytometry or microbiology as dictated by microscopy. Multiple aspirates from individual nodes from each nodal station were pooled for further analysis. The same pathologist reviewed all slides and subsequent cellblock sections, issued a diagnosis and forwarded cellblocks to molecular pathology laboratory for mutation analysis. Slides and all cellblocks were also reviewed, according to our local clinical guidelines, by panel of thoracic histopathologists and cytopathologists. Final diagnosis and disease stage [49] was agreed after discussion at the Thoracic Cancers multidisciplinary meeting. All consecutive patients undergoing EBUS-TBNA between May 2009 and February 2010 and who were diagnosed with NSCLC or were staged for their known NSCLC were included in this study. Between March 2010 and February 2011 all consecutive patients with non-squamous NSCLC were evaluated.

### Mutation Analysis

#### Sample processing and cell block preparation for DNA extraction

Three 10 micron sections from these paraffin-embedded EBUS cellblocks were deparaffinized and DNA was isolated from the tissues using Qiagen DNA isolation kit with a modified protocol. Briefly, deparaffinised tissues were suspended in 180 µ of tissue solubilizing buffer and 70 µl of proteinase K enzyme and incubated at 56°C overnight. After the addition of 200 µl of DNA binding buffer, the mixture was incubated at 70°C for 10 minutes followed by 100 µl of isopropanol and centrifugation at 16 kg for 1 minute to eliminate tissue debris. The quality of DNA and its suitability for PCR amplification was assessed by DNA-OK PCR kit, according to manufacturer's instructions.

### COLD-PCR

COLD-PCR was performed according to the principles devised by Li J *et al* [22] with minor modifications Multiple primers (eight-ten per amplicon) were designed and synthesised to amplify exons 18–21 of *EGFR* and codons 12, 13 and 61 of *KRAS*. Mutated DNA for each of the target amplicons was synthesised and serially diluted with wild type DNA to maximal dilution of 5% (mutated to wild type DNA). Standard and COLD-PCR reactions were then performed using different annealing and denaturing temperatures with each primer pair. The heteroduplex formation temperature was required to be significantly higher than the annealing temperature of the primers to avoid premature extension. We evaluated different COLD-PCR parameters and selected the best for improving sensitivity for the amplicons amplified. As some of the primer sets produced amplification at annealing temperature of up to 66°C, the heteroduplex annealing step was finally performed at 71°C. Heteroduplex denaturing temperatures between 85°C–89°C were assessed at 0.5–1°C increments before the final heteroduplex denaturing temperature of 87°C was selected. Final sensitivity of our COLD-PCR and sequencing protocol was 5–10% compared to 30% for standard-PCR and sequencing (K Tobal, unpublished data; p<0.01).

The sequences of the synthetic oligonucleotides used for the amplification of exons 18–21 of *EGFR* and codons 12, 13 and 61 of *KRAS* are shown in Table 4. Standard and COLD-PCR amplifications were performed in 50 µl reactions containing 2 mM MgCl_2_, 1 ρmole dNTPs and 2 U of AmpliTaq Gold (ABI). For the COLD-PCR amplification, DNA was first subjected to a preliminary 10 cycles of normal PCR to accumulate copies of the target sequences, followed by 40 cycles of COLD-PCR to preferentially amplify the mutant alleles and increase the sensitivity of detecting *EGFR* and *KRAS* mutations, by denaturing the double stranded PCR amplicons followed by incubation at 71°C for 3.5 minutes to produce mutant/wild type heteroduplexes. This was followed by denaturing at 87°C for 20 seconds which preferentially denatures heteroduplex amplicons thus enabling the preferential amplification of mutant DNA. PCR parameters were: 95°C for 10 minutes, then 10 cycles of 94°C for 30 seconds, 56°C for 30 seconds, 72°C for 30 seconds, followed by 40 cycles of 94°C 20 seconds, 71°C for 3.5 minutes, 87°C for 20 seconds, 56°C 30 seconds, 72°C 30 seconds. This is followed by 1 cycle of 72°C for 5 minutes. PCR parameters for standard PCR were: 95°C for 10 minutes, followed by 40 cycles of 94°C 20 seconds, 56°C 30 seconds, 72°C 30 seconds. This is followed by 1 cycle of 72°C for 5 minutes. 5 µl of PCR products were separated on 2% agarose gel to validate the amplification of the various exons. Cold and standard PCR products were then purified by Invitrogen PCR purification kit (ChargeSwitch PCR Clean-Up Kit) and sequenced in both directions using ABI 3.7 sequencing kit. Wild type DNA was used as negative control and mutated DNA for each amplicon was used as positive control in all reactions.

**Table 4:** PCR Oligonucleotide Sequences.

*EGFR*	Oligonucleotide sequence
Exon18 Forward	CATGGTGAGGGCTGAGGTGA ^5^
Exon18 Reverse	CCAGAGGCCTGTGCCAGGGAC ^5^
Exon19 Forward	CATGTGGCACCATCTCACA^5^
Exon19 Reverse	GACCCCCACACAGCAAAG ^5^
Exon 20 Forward	AAGCCACACTGACGTGCCTCT ^5^
Exon 20 Reverse	CCCGTATCTCCCTTCCCTGA ^5^
Exon 21 Forward	CCTCACAGCAGGGTCTTCTCTG ^5^
Exon 21 Reverse	TGGCTGACCTAAAGCCACCTC ^5^

*KRAS*	
Codons 12&13 Forward	TCATTATTTTTTATTATAAGGCCTGCTGAA^5^
Codons 12&13 Reverse	CAAAGACTGGTCCTGCACCAGTA ^5^
Codon 61 Forward	CAATTTAAACCCACCTATAATG ^5^
Codon 61 Reverse	CAATTTAAACCCACCTATAATG ^5^

DNA extracted from EBUS-derived aspirates from 25 of the 94-adenocarcinoma samples was analysed in parallel by cold and standard PCR amplification and subsequent sequencing of exons 18 to 21 of *EGFR* and exon 2 of *KRAS*. These samples were also analysed in parallel by COLD-PCR and SARMS (DsX *EGFR* PCR mutation analysis kit; QIAGEN), according to manufacturer instructions.

### Statistical Analysis

Categorical variables were analysed by χ^2^ or, where size was small, by means of Fischer's exact test. A Student's t test was conducted for continuous variables for comparisons between groups.
